# Effect of the COVID-19 Confinement Period on Selected Neuromuscular Performance Indicators in Young Male Soccer Players: Can the Maturation Process Counter the Negative Effect of Detraining?

**DOI:** 10.3390/ijerph19094935

**Published:** 2022-04-19

**Authors:** Nikolaos D. Asimakidis, Stylianos S. Vasileiou, Athanasios A. Dalamitros, Pantelis T. Nikolaidis, Vasiliki Manou

**Affiliations:** 1 Laboratory of Evaluation of Human Biological Performance, School of Physical Education and Sport Sciences, Aristotle University of Thessaloniki, 57001 Thessaloniki, Greece; n.asimakidis@gmail.com (N.D.A.); stelios_vas_19@yahoo.gr (S.S.V.); dalammi@phed.auth.gr (A.A.D.); vmanou@phed.auth.gr (V.M.); 2 School of Health and Caring Sciences, University of West Attica, 12243 Athens, Greece

**Keywords:** training cessation, agility, sprint, vertical jump, peak height velocity, individual responses, youth soccer

## Abstract

The COVID-19 outbreak has led to an unprecedented long-term cessation in athletes’ training routines. This study examined the effect of a 32-week detraining period, caused by the COVID-19 pandemic lockdown, on selected neuromuscular performance indicators in 29 young male soccer players, assessed close to their adolescent growth spurt (age = 13.0 ± 0.8 years). Change of direction ability of both lower limbs (COD), linear sprint times (10 and 20 m), and vertical jump height (CMJ) was evaluated twice, once before the first national lockdown, and one week after the return to training activities. Paired-sample *t*-tests detected significant improvements in all three testing variables (COD: 2.82 ± 0.23 vs. 2.66 ± 0.22 s, *p* ≤ 0.005, 0.001, effect size [ES] = 0.91 to 1.05 for the right and left limb, respectively; 10 m: 2.12 ± 0.16 vs. 1.96 ± 0.15 s, *p* ≤ 0.001, effect size [ES] = 1.67, 20 m: 3.56 ± 0.3 vs. 3.42 ± 0.27 s, *p* ≤ 0.001, effect size [ES] = 1.02 and CMJ: 23.3 ± 7.5 vs. 24.5 ± 7.6 cm, *p* = 0.033, ES = 0.42). These results indicate that maturation-related adaptations can lead to enhanced change of direction, linear sprint, and vertical jump performance, even in the absence of exposure to any level of exercise. Soccer coaches and practitioners working with youth athletes should consider the stage of maturation when planning and implementing training programs aiming to enhance neuromuscular performance.

## 1. Introduction

Soccer is an intermittent sport where the attainment of a high-performance level relies on a variety of different factors, such as psychological, physical, technical, and tactical skills [1]. From a physiological perspective, numerous high-intensity actions are performed during a 90-min soccer game, including jumping, passing, shooting, tackling, turning, sprinting, and changing pace [2]. In youth soccer, specific indicators of neuromuscular performance are, namely, change of direction, linear sprint, and jumping ability can influence match performance to a great extent [3]. Elite and sub-elite adolescent soccer players have been shown to outperform non-elite soccer players in the abovementioned neuromuscular performance indicators [4], whereas adolescent players who successfully transitioned to professional adult soccer showed higher muscle power values compared with their counterparts who did not transition to the professional level [5].

Given the popularity of soccer and the high rate of participation of young athletes, a key challenge for the practitioners involved is dealing with the complexity of growth and maturation, as developmental athletes should not be treated as “miniature adults” [6]. Biological maturation is the rate of progress toward full adult stature or a mature state, with significant interindividual differences in various biological components observed between athletes of the same chronological age across adolescence. As a result, the timing and pace of the adolescent growth spurt vary greatly between individuals [7]. Peak height velocity (PHV) is a key benchmark of maturity status, referring to the maximum rate of growth of body size [6]. Numerous physical fitness components have been shown to reach their peak development at PHV [8]. The Youth Physical Development Model developed by Lloyd and Oliver [6] proposes that the majority of physical fitness components are trainable regardless of maturity stage, although the mechanisms that underlie such training adaptations are likely to differ between childhood and adolescence.

Neuromuscular performance increases linearly with age, and even in the absence of any structured training program, remarkable increases in change of direction, linear speed, and muscle power values are typical during developmental years [7,9]. Nevertheless, young athletes who undergo age-appropriate resistance or plyometric training may display greater improvements in these performance indicators compared with their peers who did not participate in similar intervention programs [10,11,12]. Indeed, 6 weeks of plyometric training resulted in significant increases in linear speed and vertical jumping ability in young soccer players during the PHV period [13]. These findings may be indicative of “synergistic adaptation”, a term referring to the symbiotic relationship between the specific adaptations of an imposed training stimulus and the concurrent adaptations associated with growth and maturation [14], suggesting that practitioners should choose the most appropriate training protocols according to maturation stage. A recent meta-analysis [15] supported this theory, reporting that pre-PHV adolescents demonstrate greater improvements in physical fitness components with plyometric training, whereA pre-PHV and post-PHV adolescents experience greater benefits with strength training; however, in the field of pediatric exercise science, the research design and type of training interventions implemented have not been shown to differentiate between expected growth-related changes and those related to training; hence, further research using longitudinal methods is suggested. 

Coronavirus disease 2019 (COVID-19) is an infectious disease caused by severe acute respiratory syndrome coronavirus 2 (SARS-CoV-2) that spread rapidly across the world in the early months of 2020, causing thousands of cases of respiratory illness [16]. As a consequence, the World Health Organization declared COVID-19 a pandemic, imposing a state of quarantine and confinement in which exercise was restricted [17]. Apart from the social, psychological, and economical consequences [18], COVID-19 has also adversely affected sporting activities, as major competitions and events were canceled or postponed. Professional soccer has also been severely affected [19]. It is expected that when players interrupt their training, even for a short period, a substantial reduction in fitness levels will probably occur. Indeed, interruption of training can lead to partial or complete loss of training-induced adaptations in response to an inadequate training stimulus [20]. More specifically, during the COVID-19 era, several studies have shown that soccer players experienced reduced physical performance after the confinement period [19,21,22,23,24]. 

As mentioned earlier, it is difficult to accurately distinguish between training-induced and maturation-induced adaptations; however, this unprecedented situation caused by the COVID-19 pandemic could provide a unique opportunity to investigate the adaptations associated with the maturation process without undergoing any type of exercise training; therefore, the objective of this study was to examine the effect of maturation on selected neuromuscular performance indicators in young soccer players in the absence of any training stimulus due to the confinement period imposed by the second wave of the COVID-19 pandemic. Any findings could be of great importance to practitioners and sports scientists, as the “net” effect of maturation on physical fitness components, without concomitant exposure to adequate levels of exercise, would be revealed. We hypothesized that this long period of training cessation would have a detrimental effect on the explosive actions of young soccer players, as a response to the removal of any training stimulus. 

## 2. Materials and Methods

This longitudinal study aimed to investigate the effects of the lockdown implementation, due to the COVID-19 pandemic, on the performance of SSC actions in young soccer players. The suspension of the training process lasted approximately 32 weeks (128 days), and during this period, only a small amount of physical activity was possible. Moreover, no structured online training guidance was delivered. The participants were measured twice: the first testing period was just before the beginning of the first national lockdown in Greece (TP1), whereas the second testing period took place one week after the youth soccer academies resumed their operation (TP2). Change-of-direction ability of both legs (505 change of direction test), linear speed (10 m and 20 m sprint tests) and vertical jumping ability (countermovement jump—CMJ), were the neuromuscular performance indicators evaluated. Furthermore, the maturity level, expressed as years from PHV, was calculated for each athlete in both testing periods. Subjects were familiarized with the testing procedures prior to the first measurement.

### 2.1. Subjects

Twenty-nine young soccer players from the same soccer academy, aged between 12 and 14 years old, with a soccer training experience of 5.6 ± 1.2 years, volunteered to participate in this study. The descriptive characteristics of the participants are provided in Table 1. The Godin-Shephard Leisure-Time Physical Activity Questionnaire (GSLTPAQ) was used to assess the level of physical activity during the confinement period, with the results yielding an index of 7.4, classifying the activity level as insufficient [25]. During the first testing period, the subjects maintained a frequency of 3 to 4 training sessions (∼90 min session duration) per week, including a strength and conditioning session, as well as a competitive game in the regional youth championship. Subjects were injury-free during the assessment. The testing procedures were performed with the participants in an optimal nutritional and hydration state and instructed to refrain from strenuous exercise for 24 h before testing to prevent unnecessary fatigue effects. All participants and their parents were informed about the aim of the study, the procedures, the benefits, and the potential risks. Since the entire group of participants in this study was under 18 years old, signed written informed consent was obtained from all athletes and their parents before participating. All procedures were in accordance with the Helsinki Declaration and were approved by the Institutional Review Board.

### 2.2. Procedures

The TP1 was conducted for the entire group of participants, whereas during the TP2, the athletes visited the facility in subgroups (4 players were evaluated per hour), following the guidelines set by the government. Anthropometric characteristics (i.e., standing height, sitting height, and body mass) were recorded upon arrival. Based on these data, the maturity level of the participants was established. Subsequently, a warm-up routine was performed in a standardized manner, consisting of low-intensity running, low-intensity plyometric exercises, dynamic stretching, and linear and multidirectional sprints at 75% of maximal speed and maximal speed. All testing procedures were performed at the club’s facilities in the following order: countermovement jump, 10-m and 20-m sprint tests, and 505 COD speed test. To lessen the effects of fatigue on consecutive tests, 60 s to 3 min rest periods were allowed between efforts.

### 2.3. Anthropometric Measurements

Standing height and sitting height were measured to the nearest centimeter using a stadiometer (SECA 321; Vogel & Halke, Hamburg, Germany). Body mass was measured to the nearest 0.1 kg using a portable scale (Scales, SECA, 770; Vogel & Halke).

### 2.4. Estimation of Biological Maturation

The stage of biological maturation was calculated following the procedure described by Mirwald et al. [26], a non-invasive method using an estimation equation that included measures of chronological age, body mass, and standing and sitting height, to predict the years from peak height velocity. The predicting equation was: maturity offset = −9.236 + [0.0002708 × leg length and sitting height interaction] − [0.001663 × age and leg length interaction] + [0.007216 × age and sitting height interaction] + [0.02292 × body mass by height ratio].

### 2.5. Countermovement Jump

The Vertical Jumping test (CMJ) was performed using the OptoJump optical measurement system (Microgate, Bolzano, Italy)*,* which has proven to have strong concurrent validity and excellent test–retest reliability to estimate vertical jumping height [27]. For the CMJ, the participants were initially instructed to step between the optic cells while maintaining a comfortable bilateral stance with hands supported at the hips. When they felt ready, they executed a countermovement by flexing their hips and knees to a self-selected depth, and then accelerated vertically into a jump as fast as possible. Jumping height was measured in centimeters (cm). Three CMJ testing trials were performed, with the best effort being used for the analysis. A rest interval of 60 s was allowed between each attempt. If the players released their hands from their hips, the jump was considered invalid and was repeated after a 60-second pause. The intraclass correlation coefficient (ICC) for test-retest reliability was 0.97.

### 2.6. 10 m and 20 m Linear Sprint Test

The times for the 10 m and 20 m linear sprint tests were recorded using timing gates (WITTY System, Microgate), positioned at 0, 10, and 20 m, so that the 0–10 m and 0–20 m splits could be measured during a single effort, at a height of 1 m. Participants were instructed to adopt a standing start position with the forward foot 0.3 m behind the first gate to inhibit any premature tripping of the first gate. Subjects were free to choose the foot placement during the start. Moreover, they were instructed to sprint maximally through all timed gates upon beginning their effort. Time was recorded to the nearest 0.001 s. Each subject completed 3 trials, with 3 min of recovery time allowed between trials. The fastest 0–10 and 0–20 m splits were used for further analysis. The ICCs for test-retest reliability were 0.77 and 0.87 for the 10-m and 20-m sprint respectively.

### 2.7. 505 Change of Direction Test

As it has been previously used in soccer, the 505 test was the chosen testing procedure, performed as previously described in detail, to evaluate the change of direction ability [28]. Each subject completed 2 trials for each limb, with 3 min of recovery between trials, in a randomized order. The fastest trial for each leg was used for further analysis. The ICC for the dominant and non-dominant leg was 0.72 and 0.70, respectively.

### 2.8. Statistical Analyses

Mean values and SDs were used to describe the data. All data were checked for normality using the Shapiro–Wilk test. Paired-sample t-tests were used to compare the differences between the pre-test and post-test measurements. The magnitude of the difference between group means was calculated by Cohen’s d effect sizes using the following formula: (*Meanposttest–Meanpretest*)/*SD*, and classified as trivial (<0.2), small (0.2–0.6), moderate (0.6–1.2), large (1.2–2.0), or very large (2.0–4.0) [29]. The variability in the responses to the detraining period was evaluated using the coefficient of variation (CV), (i.e., the ratio between the standard deviation and the mean, expressed as %) [30]. The smallest worthwhile change (SWD) was calculated as 0.2 × between-subject SD [31], expressed as a percentage. Percentage changes were calculated as ([TP2 value–TP1 value]/TP1 value) × 100. The level of significance was set at *p* ≤ 0.05. The test–retest reliability for each test was assessed using the ICC.

## 3. Results

**Figure 1:**
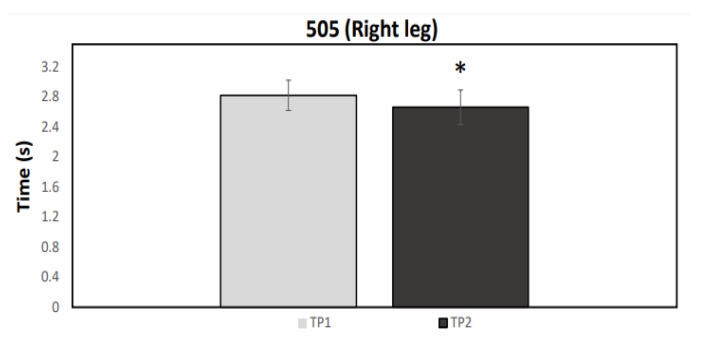
Mean values for the 505 COD test performance for the right leg during the two testing points (TP1 and TP2). * Statistically significant difference (*p* < 0.05).

**Figure 2:**
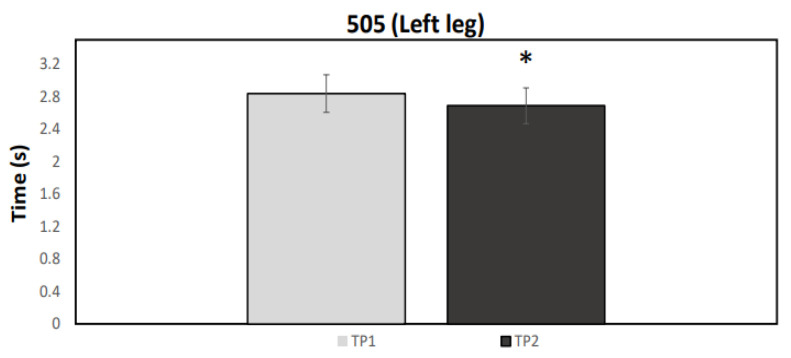
Mean values for the 505 COD test performance for the left leg during the two testing points (TP1 and TP2). * Statistically significant difference (*p* < 0.05).

**Figure 3:**
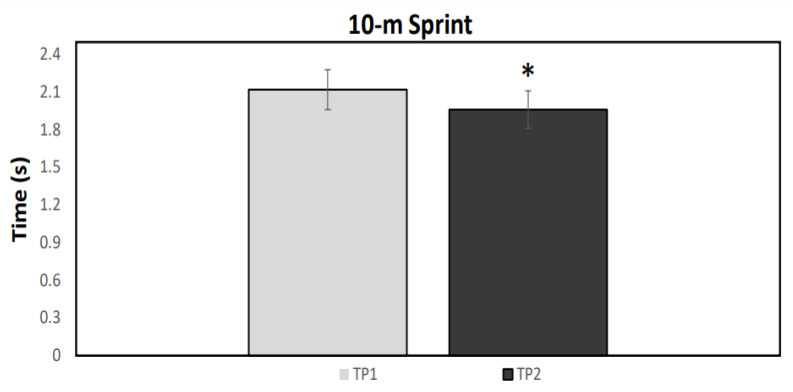
Mean values for the 10-m linear sprint performance during the two testing points (TP1 and TP2). * Statistically significant difference (*p* < 0.05).

**Figure 4:**
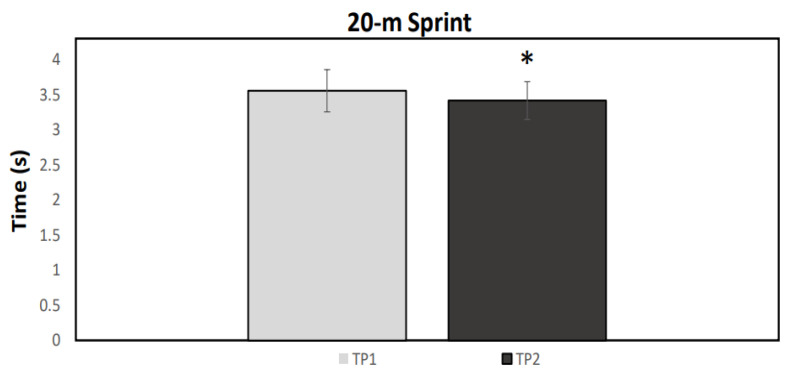
Mean values for the 20-m linear sprint performance during the two testing points (TP1 and TP2). * Statistically significant difference (*p* < 0.05).

**Figure 5:**
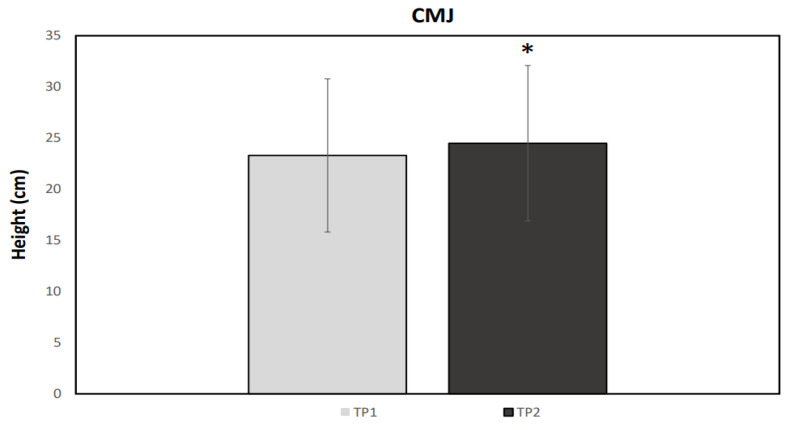
Mean values for the CMJ test performance during the two testing points (TP1 and TP2). * Statistically significant difference (*p* < 0.05).

**Figure 6:**
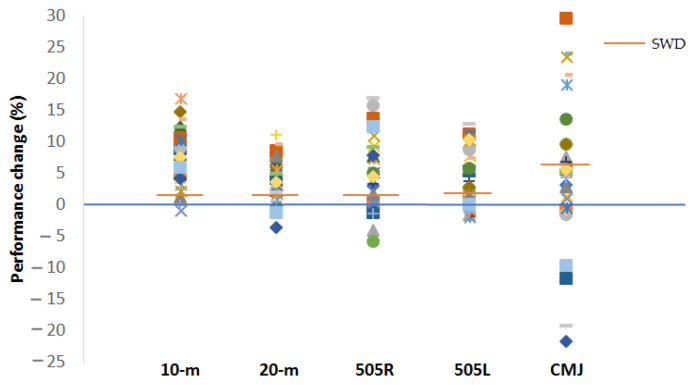
Individual percentage changes and smallest worthwhile changes for all five performance tests during the two testing points (TP1 and TP2). Each symbol represents the percentage change for each participant whereas the orange line indicates the percentage of the smallest worthwhile change for each test. Note: 505R = change of direction test for turns of the right leg; 505L = change of direction test for turns of the right leg; SWD = smallest worthwhile change (0.2 × between-subject SD).

A normal distribution was observed for all data (*p* > 0.05). Figure 1, Figure 2, Figure 3 and Figure 4 illustrate the group data for COD ability and linear sprint, respectively. Significant differences were observed for COD ability, using both the right (*p* = 0.037, ES = 0.91, 5.43 ± 5.98%) and the left limb (*p* = 0.007, ES = 1.05: moderate effect, 5.22 ± 4.86%) (Figure 1 and Figure 2). Similarly, 10 m (*p* < 0.001, ES = 1.67: large effect, 7.85 ± 4.52%) and 20 m sprint times (*p* < 0.001, ES = 1.02: moderate effect, 3.73 ± 3.55%) were significantly enhanced from TP1 to TP2 (Figure 3 and Figure 4). Finally, a significant difference was also found in CMJ height (*p* = 0.033, ES = 0.42: small effect, 4.96 ± 11.75%) after the 32-week period (Figure 5). Individual percentage responses during TP1 and TP2 showed a slight change in all performance tests (CV = 32.02 vs. 31.41%, 7.67 vs. 8.09%, 8.53 vs. 8.15%, 7.34 vs. 8.64%, and 8.13 vs. 8.30%, for the CMJ, 10 m, 20 m, and COD test of the right and left leg, respectively) (Figure 6). The smallest worthwhile change to indicate meaningful improvement in performance was 1.5% (0.03 s), 1.7% (0.06 s), 1.5% (0.04 s), 1.6% (0.05 s), and 6.6% (1.5 cm), for the 10 m, 20 m, COD of the right leg, the left leg, and the CMJ, respectively (Figure 6).

## 4. Discussion

The aim of this study was to examine the effect of biological maturation on selected neuromuscular performance indicators in young soccer players in the absence of any training stimulus. Change of direction ability, 10 m and 20 m sprint, and CMJ were all significantly improved after the training cessation period, implying that maturation-related adaptations can lead to enhanced neuromuscular performance, even in the absence of exposure to sufficient levels of exercise in young soccer players.

The results of the 505 COD test showed a significant improvement in COD ability for both limbs (5.43% and 5.22% for the right and left limb, respectively) after the confinement period. To the best of our knowledge, this is the first study to examine the effects of a COVID-19 lockdown period on COD ability in young soccer players. Previous research on young soccer players has demonstrated that COD ability reaches its peak development at the age of 13 to 14 years old, which is consistent with the PHV [8,32]. Circumpubertal and post-pubertal adaptations are likely mediated by increases in sex androgen concentrations such as testosterone, growth hormone, and an insulin-like growth factor [33]. Such hormonal changes produce an increased force-generating capacity that results from continued neural development as well as increases in muscle cross-sectional area, muscle pennation angle, and continued fiber-type differentiation [32]. According to Young et al. [34] force-generating capacity is associated with COD performance, potentially explaining the underlying mechanisms that resulted in the improvements observed in our study.

Regarding the linear sprint performance, 10 m and 20 m sprint times were significantly improved by 7.85% and 3.73%, respectively, after the confinement period. These findings are in contrast to the results of similar studies that also investigated the effects of the COVID-19 lockdown on sprint performance in soccer [21,23,24] and futsal [35]. More specifically, Alvurdu et al. [21] reported significant decrements in 30 m sprint performance after 109 days of training cessation, whereas, similarly, Grazioli et al. [23] showed that 63 days of home confinement led to a reduced 10 m and 20 m sprint performance in professional soccer players. In the study by Spyrou et al. [35] the same results were observed in elite futsal players, as 70 days of reduced training impaired 10 m sprint performance. By contrast, the study conducted by Korkmaz et al. [24] examined the effect of 89 days of detraining due to the COVID-19 confinement period in semi-professional soccer players, demonstrating no significant differences in 30 m sprint performance; therefore, pre-detraining status may impact the magnitude effect of a long-term training cessation period. Moreover, taking into account the fact that the maturity stage of our sample was very close to the emergence of PHV (−0.1 years before PHV), it may be suggested that the different results presented here could be a consequence of the adolescent growth spurt, as sprinting ability reaches its peak development at PHV as previously shown [8], possibly due to an increase in stride length due to greater propulsive forces and leg stiffness [36], both stemming from maturation-related adaptations.

Jumping ability, assessed through the CMJ performance, also showed a significant increase (4.96%). Previous research attempting to investigate the effects of the COVID-19 confinement period on vertical jumping performance has yielded equivocal results [23,35,37,38,39,40]. In the studies by Spyrou et al. [35] and Cohen et al. [37] in elite futsal and soccer players, respectively, CMJ height values remained unchanged, yet differences in eccentric and landing phase kinetic variables were observed. Additionally, in a soccer study [40], no differences in CMJ height values were noted, although decreased peak CMJ power output values in elite soccer players were observed. On the contrary, Grazioli et al. [23] demonstrated decreased CMJ height values in professional soccer players, whereas Parpa and Michaelides [38] and Pucsok et al. [39] reported similar results to those presented here in professional and adolescent soccer players, respectively; however, both studies employed a home training protocol consisting of bodyweight exercises (including plyometric exercises), thus potentially masking the net effects of training cessation. Moreover, changes in the neuromuscular system during growth and maturation (specifically, increases in muscle size, pennation angle, fascicle length, tendon stiffness, and motor unit recruitment) have been reported to lead to enhanced performance related to explosive actions [41]. The inclusion of an additional CMJ variables assessment should probably also be investigated, as variables other than CMJ height are more sensitive to changes (e.g., eccentric and concentric phase duration) [42], a finding that has also been confirmed in some of the abovementioned studies [34,37,40].

Identifying individual responses to training or detraining is of great importance in sports [30], especially when young athletes are involved. The smallest worthwhile change, a statistical value calculated by multiplying the between–subject *SD* value by 0.2 for team sports [31], indicates meaningful or a “real” performance improvement related to game situations (e.g., two players through a sprint duel). To monitor individual responses in team sports, pre-defined values corresponding to ~1.0% (0.04 s to 0.06 s) for 10 m to 40 m sprints and ~2.5% (1.0 cm) for the CMJ height have been previously suggested [43,44]. In this sense, for the entire group of participants, regarding the 505 change of direction test, 22 participants demonstrated a meaningful performance enhancement for the right leg, and 19 for the left leg. For the 10 m and 20 m linear sprint performance, 27 and 21 participants demonstrated a “real” improvement, respectively. Finally, 11 participants showed a meaningful CMJ improvement. 

According to the youth physical development model [6], COD ability, linear speed, and muscle strength are trainable throughout adolescence; therefore, it is recommended that practitioners should be aware of the “windows of opportunity” that enable them to take advantage of the trainability of these physical components due to maturation-related adaptations. More specifically, a key period for the development of muscle strength is the onset of adolescence, a period in which androgen concentrations, and consequently muscle mass, increase. Concerning linear speed, the training focus for preadolescent athletes should be high neural activation (plyometrics and sprinting), whereas, for adolescent athletes, neural and structural development (i.e., strength and plyometrics) should represent the primary emphasis of training. Ultimately, as force-generating capacity and linear speed are associated with COD ability [33], prepubertal athletes should focus on taking advantage of neural adaptations that contribute to the development of sprint speed, whereas adolescent athletes should aim to capitalize on the increases that occur in peak force. Although a recent review has questioned the potential usefulness of the windows of opportunity to optimize training adaptations in young athletes [45], it seems that more research is required in this field.

Some limitations should be noted when interpreting the results of this study. A sample including an additional group of more “mature” players (i.e., post-PHV) might have provided useful information on how the training cessation period affected maturing soccer players and might have allowed a comparison between two different maturity groups (pre-PHV vs. post-PHV). In addition, some more “sensitive” CMJ variables (e.g., time of flight to contraction time) would have allowed a more holistic interpretation of the effects of the COVID-19 confinement period on CMJ performance. 

Based on our results, a long-term (32 weeks) training cessation period due to COVID-19 quarantine not only failed to impair explosive performance as initially hypothesized, but, on the contrary, resulted in improvements in selected neuromuscular performance indicators. Importantly, homogeneous results regarding the individual responses were obtained, since the majority of the participants exhibited a higher performance, especially during the linear sprint tests; hence, certain important practical recommendations can be derived for practitioners working with young soccer players. Firstly, after a long-term training cessation, other physical performance indicators (e.g., cardiorespiratory endurance) should be assessed and trained, besides those evaluated here; however, given the marginal improvements observed in CMJ height after the training cessation, it would be prudent to include some supplementary assessments (i.e., CMJ kinetic variables) to determine that vertical jump performance has indeed improved, as well as to obtain a more holistic view of the effects of long-term training cessation on vertical jump performance in developmental athletes. Secondly, practitioners should anticipate that improvements in neuromuscular performance in athletes undergoing the adolescent growth spurt may result solely from maturation-related adaptations, leading to ambiguity as to whether their training intervention is considered successful. They should therefore plan and implement training programs that are favorable to this stage of maturation, to capitalize on the synergistic adaptation. Importantly, the increase of androgens, such as testosterone, at this stage of development, could allow practitioners to employ more advanced methods of strength training (e.g., combined training), always backed up with the relevant assessment of technical competency in order to promote further adaptations. 

## 5. Conclusions

In summary, a 32-week period of training cessation resulted in significant improvements in COD, 10 m and 20 m sprint, and CMJ values in young male soccer players around the age of the peak pubertal growth spurt. This may suggest that even in the absence of an adequate training stimulus, maturation-related adaptations are capable of enhancing exercise performance-related neuromuscular performance. The information presented here could provide practitioners and sports scientists with clear evidence, suggesting that a long period of training cessation close to the adolescent growth spurt may not necessarily result in reductions in selected performance indicators, and any exercise performance improvements during the developmental years may be solely attributed to maturation-related adaptations.

## Figures and Tables

**Table 1:** Descriptive characteristics of the participants during the two testing periods.

Variables	TP1	TP2	Mean Difference Pretest-Posttest (95% CI)
Age (years)	13.0 ± 0.8	13.7 ± 0.7 *	0.7 (0.6–0.7)
Height (cm)	163.5 ± 7.9	168.6 ± 6.8 *	5.1 (3.9–6.1)
Body mass (kg)	55.3 ± 11.4	58.7 ± 11.2 *	3.4 (1.9–4.9)
Maturity offset (years)	−0.8 ± 0.9	−0.1 ± 0.8 *	0.7 (0.6–0.8)

Note: TP1 = Just before the beginning of the first national lockdown; TP2 = one week after the youth soccer academies resumed their operation; * significant difference between the two testing points (*p* < 0.05).
